# From Geometry to Flow Allocation: A Physics-Based Framework for Interpretable Microvascular Hemodynamics

**DOI:** 10.3390/bioengineering13091091

**Published:** 2026-09-20

**Authors:** Alexander Fiedler, Marius Drysch, Sonja Verena Schmidt, Pia Weskamp, Felix Reinkemeier, Flemming Puscz, Alexander Sogorski, Marcus Lehnhardt, Christoph Wallner

**Affiliations:** Department of Plastic Surgery and Hand Surgery, Burn Centre, BG University Hospital Bergmannsheil, Ruhr University Bochum, Bürkle-de-la-Camp-Platz 1, 44789 Bochum, Germany

**Keywords:** end-to-side anastomosis, two-dimensional modeling, flow allocation, signed wall shear, lattice Boltzmann method, computational hemodynamics, controlled numerical experiment

## Abstract

Anastomotic angle and flow allocation change together in end-to-side junctions, complicating interpretation of angle-dependent wall shear. We used MITOS Flow Lab, a two-dimensional D2Q9 two-relaxation-time lattice-Boltzmann environment, to examine this coupling in steady, rigid-walled, Newtonian models at Re ≈ 91. Angles of 30–120° were compared under equal outlet pressures and at approximately matched branch-flow fractions of 0.25 and 0.21, achieved with angle-specific static outlet-pressure offsets. Under equal outlet pressures, the branch-flow fraction decreased from 0.356 to 0.151 across this angle range, while the minimum normalized signed recipient-floor shear increased from 0.140 to 0.450. Matching flow allocation substantially reduced angle-associated variation in this endpoint and in the sub-toe response, whereas sub-heel and sub-ostial responses remained angle dependent under the adjusted boundary conditions. This qualitative contrast persisted when the lumen resolution was increased from 32 to 64 nodes for the 0.25 target, although minimum-shear attenuation changed from approximately 83% to 74%. The 0.21 target was examined only on the production grid. These experiments demonstrate that the interpretation of angle-associated shear depends on the flow-allocation condition used for comparison. They do not identify a boundary-independent geometric effect or a causal mediation fraction. Absolute values and attenuation magnitudes remain sensitive to discretization and have not been independently validated. These controlled comparisons provide a framework for interpreting angle-associated shear together with achieved flow allocation and the specified outlet conditions.

## 1. Introduction

Arterial end-to-side anastomosis is commonly used in reconstructive microsurgery when the recipient vessel must be preserved, when a single remaining runoff vessel makes sacrifice undesirable, or when donor and recipient calibers differ substantially. The anastomotic angle is readily visible and directly controlled during construction, and it has therefore been a frequent focus of technical and hemodynamic studies.

Published studies have not identified a consistent preferred angle. In a rat carotid model, Zhang et al. [1] found that at thirty minutes the grafted vessel received 56.5% of total carotid flow at 45°, 46.5% at 90°, and 43.2% at 135°, a difference no longer apparent at two hours. Zoubos et al. [2], comparing arteriotomy shape and angle in rat iliac arteries, found no difference in patency and recommended a 90° elliptical arteriotomy on technical grounds. In vivo measurements confirmed that anastomotic angle changes the flow field at end-to-side junctions [3], while bench measurements showed an effect on the hydraulic resistance of the connection itself [4]. Computational studies have likewise produced differing results: Jinka et al. [5] reported increasing recirculation with angle, with minima at 30° and 45°, whereas Wongchadakul et al. [6] reported the lowest maximum wall shear rate at 90°. Parameterized computational work has also demonstrated the importance of suture-level geometry and pulsatility in microarterial anastomoses [7,8,9]. Recent studies have examined stenotic coronary end-to-side anastomoses [10], venous anastomotic angles and graft configurations in arteriovenous grafts [11], and experimental flow fields in an end-to-side junction [12].

Computational bioengineering provides a way to investigate such coupling through explicitly defined comparisons. Coronary bifurcation studies have examined how angulation, vessel caliber, and related morphology influence local flow and wall-shear distributions [13,14,15]. These studies establish a broader geometry–hemodynamics context; their specific shear patterns depend on the vascular configuration and boundary conditions. Computational flow analysis has also been applied to the morphological and functional assessment of surgically constructed anastomoses during skills training [16]. The present contribution is to compare angle series at approximately matched flow allocation within a fixed two-dimensional modeling framework.

A plausible contributor to divergent angle findings is that changing angle can alter both junction shape and the pressure–flow relationship of the two outflow paths. Zhang et al. [1] provide experimental evidence for angle-dependent allocation, while Leva and Engström [4] demonstrated angle-dependent connection resistance. Hydraulic-network analysis describes the contribution of distributed viscous resistance [17] and has been used to redesign microfluidic branches to equalize flow [18]. At moderate Reynolds numbers, however, junctional inertia can introduce angle-dependent nonlinear pressure–flow behavior [19]. Recent bypass modeling has examined how the graft-to-native flow ratio affects downstream flow patterns [20], while resistance–capacitance outlet models have been analyzed as a means of controlling flow-rate ratios in arterial networks [21]. The present Re ≈ 91 system should therefore not be interpreted as a purely linear resistance network. Adjusting outlet pressures to obtain a similar branch fraction provides a comparison conditional on flow allocation, while also changing the pressure field.

We developed the MITOS Flow Lab to study these relationships in a parameterized two-dimensional environment. This study describes the implementation and its numerical verification, then compares angle-associated shear responses under equal outlet pressures and approximately matched branch-flow fractions. We hypothesized that flow matching would substantially reduce angle-associated variation in recipient-floor and sub-toe shear, while a larger angular dependence would remain near the heel and ostium. Any remaining difference is interpreted as geometry associated under the specified outlet conditions, not as an independently identified geometric effect.

## 2. Materials and Methods

### 2.1. MITOS Flow Lab: Computational Architecture

MITOS Flow Lab has an interactive interface and a headless pathway for scripted batch analysis, both using the same numerical core. The platform integrates parameterized planar geometry, a D2Q9 two-relaxation-time lattice-Boltzmann solver, a clinical-unit input interface, residual monitoring, and export of fields and descriptors. The present work evaluates the stationary two-dimensional implementation.

The interface exposes anastomotic angle, heel and toe transition radius, branch-to-recipient caliber ratio, recipient diameter, systolic and diastolic pressure, heart rate, venous back pressure, a lumped resistance input, static outlet-pressure bias, and steady or pulsatile inflow. Quantitative endpoints in this study were flow allocation, signed recipient-floor shear, regional shear indices, and velocity-based recirculation. The capped residence-time display was retained for qualitative visualization only. Pulsatile and exploratory deposition functions were disabled for production analyses.

**Figure 1:**
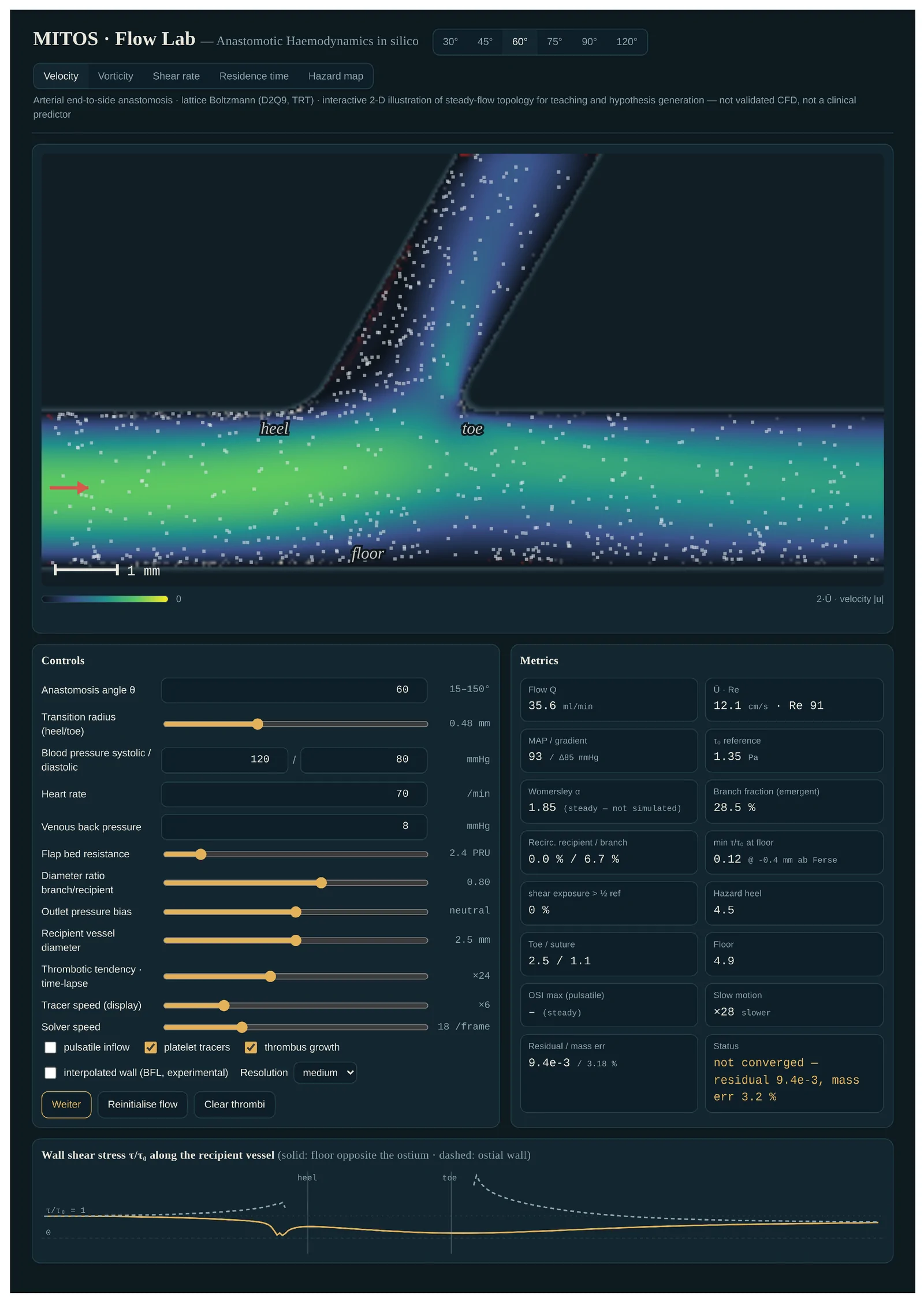
MITOS Flow Lab computational environment developed for parameterized analysis of arterial end-to-side microanastomoses. The interface allows adjustment of junction geometry and hemodynamic boundary conditions and visualizes velocity, vorticity, shear rate, residence time, and quantitative flow metrics. All production analyses were performed with the same numerical core in headless batch mode rather than by manual interaction with the interface. The configuration shown is illustrative: pulsatile inflow and exploratory tracer/deposition features are enabled in the screenshot but were disabled for all production analyses.

The platform exports run-level descriptors together with spatial wall and field data in machine-readable form. Each output remains traceable to explicit geometric and hemodynamic inputs, enabling controlled mechanistic analysis. The interactive interface (Figure 1) was used for exploration and visualization. All quantitative analyses were performed with the identical numerical core in headless batch mode using scripted parameter files, and outputs were exported directly for analysis; no numerical result in this manuscript was read from the interface. MITOS Flow Lab is a research environment for controlled in silico experiments rather than clinical decision-support software or a patient-level predictor.

### 2.2. Numerical Solver

Flow was solved on a two-dimensional D2Q9 lattice using a two-relaxation-time collision operator, with Λ = 3/16 and a halfway bounce-back at rigid walls. This magic parameter is associated with viscosity-independent wall positioning for ideal halfway bounce-back configurations; it does not establish accuracy at oblique, staircased junction walls. Blood was Newtonian, with dynamic viscosity 3.5 mPa·s and density 1050 kg m^−3^. Production runs used 174 × 107 nodes, with 32 across the recipient lumen. The mean lattice velocity of 0.06 corresponds to a mean Mach number of 0.104 and a parabolic inlet peak of 0.156. The formulation is weakly compressible; neither a lower-Mach comparison nor an independent incompressible junction benchmark is included. These are distinct from the verification checks in Section 2.8. The wider role and limitations of lattice-Boltzmann methods in microcirculatory modeling have been reviewed previously [22]. Recent vascular LBM studies have examined numerical and boundary-condition uncertainty [23] and compared arterial flow simulations with conventional CFD methods [24].

### 2.3. Geometry

The fluid domain is the union of two signed distance fields, a recipient slab and an obliquely oriented branch slab, combined with an exact circular fillet of adjustable radius at the re-entrant heel and toe corners and intersected with the floor half-space. The transition radius parameter f maps to a physical radius r = 0.55·f·D, where D is the recipient lumen diameter; the settings used correspond to r/D = 0, 0.193 and 0.385, i.e., 0, 0.48 and 0.96 mm at D = 2.5 mm.

Fluid nodes with fewer than three fluid neighbors, which arise where an oblique wall is discretized on a Cartesian lattice, are removed iteratively. Outlet nodes at the staircased edge of the branch port are treated as walls because the Zou-He condition presumes a straight boundary with fluid on both tangential sides. The branch stub length is capped so that its outlet never reaches the right-hand boundary.

### 2.4. Hemodynamic Prescription

Clinical-unit inputs were used to select a velocity scale, rather than to impose a measured pressure field throughout the computational domain. Mean arterial pressure was calculated as MAP = P_dia_ + (P_sys_ − P_dia_)/3, the nominal bed driving pressure as ΔP_bed_ = MAP − P_venous_, and the associated cylindrical flow estimate as Q_cyl_ = ΔP_bed_/R. The mean velocity U_phys_ was obtained from Q_cyl_/(πD^2^/4), and Re = ρ_phys_ U_phys_ D/μ. Baseline inputs of 120/80 mmHg, venous pressure 8 mmHg, D = 2.5 mm, and R = 2.4 mmHg·min·mL^−1^ give Q_cyl_ ≈ 35.6 mL min^−1^, U_phys_ ≈ 0.121 m s^−1^, and Re ≈ 91. Resistance inputs of 7.3 and 1.45 yield Re ≈ 30 and 150. The simulated planar flux is per unit depth; Q_cyl_ is an input-scaling convention, not a three-dimensional flow computed by the solver. The nominal 85.3 mmHg bed pressure difference must not be equated with the pressure drop across the short modeled junction.

### 2.5. Boundary Conditions and Control of Flow Allocation

The inlet uses a Zou–He parabolic velocity boundary. At the two outlets, the existing bias parameter b sets equal and opposite lattice pressure offsets after the initialization ramp: the branch offset is +4 × 10^−4^b and the distal offset is −4 × 10^−4^b, with −1 ≤ b ≤ 1. The corresponding outlet densities are 1 + 3δp_b_ and 1 + 3δp_d_. Negative b favors branch outflow; positive b favors distal outflow. Each production calculation uses a constant final offset without dynamic feedback. Thus, the branch fraction remains an output of the flow solution.

For matching, the fixed bias was selected offline by running independent constant-offset simulations. Target branch fractions were 0.25 and 0.21, with a prespecified tolerance of ±0.015. The achieved fractions for the four matched angles are reported in Section 3.3. The common attainable target range was approximately 0.204–0.252; the achieved fractions in the accepted matched comparisons ranged from 0.197 to 0.253. This is an approximately matched numerical control experiment. Changing b also changes the pressure field and can influence upstream and junctional shear; the design does not isolate a geometric effect independently of outlet treatment.

Pressure scaling follows the same physical velocity mapping as the solver. With C_u_ = U_phys_/U_LB_ and reference lattice density one, the pressure factor is C_p_ = ρ_phys_ C_u_^2^. At baseline, C_u_ ≈ 2.012 m s^−1^ and C_p_ ≈ 4.25 × 10^3^ Pa per lattice-pressure unit. The branch-minus-distal pressure difference is therefore Δp_bd_ = C_p_(δp_b_ − δp_d_) ≈ 3.40b Pa = 0.0255b mmHg. This is a conversion of the existing boundary offsets, not an additional simulation. The imposed outlet densities differ from one another by at most 0.12% each; this bound does not quantify density variation inside the domain.

The maximum outlet difference is small relative to the nominal 85.3 mmHg bed driving pressure used to set inflow. That comparison does not show that the intervention is small relative to the junction pressure drop, which was not reported. The outlet pressures are independently tunable numerical controls with equal and opposite offsets, not independently adjustable physiological measurements. A calibrated vascular-bed model would require resistance or impedance data and appropriate coupling, as illustrated by coupled coronary models [25]. Coupled lumped-parameter and lattice-Boltzmann modeling has also been applied to patient-specific aortic bypass hemodynamics [26]. No physiological plausibility claim is based on the small absolute offset alone.

### 2.6. Convergence and Acceptance

Acceptance criteria were specified before the production matrix was run. A simulation was accepted as steady only when all four criteria were met: a smoothed normalized velocity residual below 10^−5^, a smoothed boundary mass-balance error below 1%, no stability-guard reset, and a change in branch flow fraction of less than 10^−3^ over the preceding 4000 steps. No fixed iteration count was used. Runs that failed to meet these criteria within 1.4 × 10^5^ steps were excluded from quantitative analysis according to this predefined rule.

### 2.7. Wall Regions and Hemodynamic Features

Metrics and wall regions were specified in code before the final production simulations. Position along the floor wall, opposite the ostium, is expressed in recipient diameters D from the heel; the toe is located at x_toe_ = ratio/sin θ. Five regions were prespecified (Table 1):

Primary endpoints were the branch-flow fraction φ_b_, defined as branch-outlet flux divided by inlet flux; velocity-based recipient and branch recirculation; minimum normalized signed recipient-floor shear; and the signed shear-deficit burden B_low_. Recirculation was the fraction of regional nodes with velocity projected onto the local vessel axis below −0.015U_0_. Projection onto the vessel axis is necessary for an oblique branch. B_low_ retains its original computational definition but is described as a signed shear-deficit burden, rather than a biological low-shear exposure measure. Residence-time output is excluded from the quantitative mechanistic argument.

The local-flow-normalized shear ratio D_s_ is the regional mean of τ_s_/τ_0_ divided by 1 − φ_b_ for regions at or beyond the ostium. The proximal region is normalized by one. In an unchanged-width, fully developed planar channel, wall shear scales linearly with carried flux, so the analytical downstream reference is (1 − φ_b_)τ_0_. D_s_ = 1 therefore denotes this reference, not a numerically demonstrated downstream plateau. The distal window begins one diameter beyond the toe and ends three diameters beyond it; departures from unity can include junction disturbance, incomplete redevelopment, wall-discretization bias, and finite-domain or outlet effects. Moreover, applying the distal-flow denominator beneath the heel or ostium is a comparative normalization, not a claim that the local section already carries fully developed distal flow. D_s_ does not estimate a geometry-only contribution.

Wall shear is a signed component of tangential wall traction. Let ψ be the signed-distance field, negative in the fluid, and *n* its normalized gradient pointing out of the fluid. The reference axis a is fixed by geometry: the positive recipient axis on recipient walls and the branch axis on branch walls. The following equations describe the non-equilibrium-moment stress recovery, tangential projection, and normalization used in the archived implementation.

$$\Pi^{neq}=\Sigma ᵢ\;c_{i}\;⊗\;c_{i}\;(f_{i}-{f_{i}}^{eq}),\;E*=-\frac{{3\omega }_{+}}{2}\Pi^{neq}$$

$$\sigma_{v,LB}=2\nu_{LB}E*$$

Here Π^neq^ is the non-equilibrium second moment, ω_+_ is the even relaxation frequency, and *ν,_LB_* is the lattice kinematic viscosity. The implementation uses reference lattice density one in the stress recovery. The minus sign defines traction exerted by the fluid on the wall. The pressure part makes no contribution after tangential projection. At curved or oblique walls, projecting onto a vessel axis need not equal the traction magnitude.

$$n=\frac{𝛻\psi }{\left\|𝛻\psi \right\|},\;t_{\tau }=-(I-n\;⊗\;n)\sigma_{v,LB}\;n$$

$$\tau_{s}=a\;\cdot \;t_{\tau },\;\tau_{mag}=\|t_{\tau }\|$$

$$\tau_{stored}\;\leftarrow \;0.97\tau_{stored}+0.03\tau_{s}$$

The stored wall value is exponentially smoothed with weight 0.03 per update. Sign changes are retained; no absolute value is taken before the minimum or burden calculation. Thus, negative τ_s_ contribute more than one to the burden integrand and represent reversed signed traction, not simply low shear magnitude. Absence of a velocity-based recirculation criterion is not used to redefine signed shear as a magnitude.

$$\tau_{0,LB}=\frac{6\nu_{LB}U_{0}}{H},\;\tau_{0,phys}=\frac{6\mu U_{phys}}{D}\;\approx \;1.01\;Pa$$

The normalization τ_0_ is the analytical planar-Poiseuille value, not the interface’s cylindrical shear readout. Its physical equivalent is 6μU_phys_/D ≈ 1.01 Pa at baseline; the cylindrical expression 8μU_phys_/D ≈ 1.35 Pa is not used to interpret the normalized floor-shear results. In the equations, H is the number of lattice spacings across the lumen, and L_w_ is the sampled wall length.

$$B_{low}=\frac{1}{L_{w}}\;\int \;max(0,\;1-\tau_{s}/\tau_{0})\;ds,\;\tau_{min}=min(\tau_{s})$$

$$D_{s}=\frac{\langle \tau_{s}/\tau_{0}\rangle }{1-\varphi_{b}}$$

The displayed residence-time field is an Eulerian age surrogate updated by first-order upwind advection, a unit aging source, and an explicit discrete smoothing term. It is initialized to zero, reset to zero at the inlet, and clipped to the interval 0–6T, where T = D/U_0_. In lattice units, the update is a_new_ = clip[a + 2 − 2(u·∇_up_ a) + 0.12Σ_neighbors_(a_neighbor_ − a), 0, 6T], performed every second flow step. This corresponds to numerical diffusion of 0.06 lattice units for that update. Recirculating or poorly flushed regions can saturate; the field is not a Lagrangian distribution of exit times. Because the original aggregation rule and fraction of saturated samples have not been established from the run exports, medians of this field are not used for quantitative inference.

### 2.8. Numerical Verification and Resolution Robustness

Automated inlet checks compared the undisturbed profile with planar Poiseuille flow. Peak velocity agreed within 0.02%, and normalized signed shear was +0.966 on the floor and +0.991 on the ceiling. A fixed-inlet-orientation comparison across 30–150° showed upstream shear variation of at most 2.1%. These checks assess the inlet profile and sign convention; they do not validate junctional shear, oblique-wall accuracy, or pressure-outlet behavior. Mass balance and velocity-residual convergence were monitored separately. In other LBM implementations, image-based pulsatile wall-stress calculations have been compared with analytical solutions and laboratory measurements [27].

A three-grid study at 27, 36, and 48 nodes across the recipient, each run to a residual below 4 × 10^−6^, gave indicative fine-grid Grid Convergence Indices of 3.1% for minimum floor shear, 7.4% for sub-heel shear, and 1.0% for branch fraction. Observed orders of 0.4–2.4 did not establish an asymptotic regime. These indices are not formal uncertainty bounds and do not directly quantify error at the production resolution of 32 nodes, which was not part of that three-grid sequence.

A separate comparison included the production grid directly: the four angles, 30°, 60°, 90°, and 120°, were evaluated under equal outlet pressures, and the approximately 0.25 matched target at both 32 and 64 lumen nodes. These are eight conditions at each grid, giving sixteen reported condition–grid results. No complete refined-grid series is reported for the 0.21 target. Exploratory calculations at 96 and 128 nodes do not provide a documented multi-angle accuracy assessment and are not used as evidence of grid independence. Agreement in qualitative behavior is distinguished from convergence of absolute values or attenuation magnitudes.

### 2.9. Descriptive Low-Dimensional Analysis

The simulations are deterministic and are not independent biological replicates; no inferential statistics were applied. Angle sensitivity is the range of a positive endpoint across the four common matched-design angles divided by its mean. Attenuation is one minus matched sensitivity divided by equal-pressure sensitivity, expressed as a percentage; negative attenuation denotes an increased normalized range after matching. This is a descriptive comparison statistic, not a causal mediation fraction. Ordinary least-squares models summarize associations between B_low_, angle, and branch fraction within the reported simulation pool. Their R^2^ values do not demonstrate prediction, causal dominance, or generalization beyond that pool.

## 3. Results

### 3.1. Computational Implementation and Simulation Accounting

The study records report 62 simulations across production, matching, and bias exploration, of which 57 met the acceptance criteria. The production analysis comprised 26 accepted runs; the reported pooled descriptive analysis contained 40 accepted runs, including matching experiments. These summaries do not by themselves provide a complete run-level inventory. Of the five non-accepted runs, three were described: one exceeded the residual threshold (5.7 × 10^−5^ versus 10^−5^), one exceeded the mass-balance threshold (1.28% versus 1%), and one 30° configuration at caliber ratio 1.00 did not reach steady state. The parameter assignments of the first two runs and the identities of the other two non-accepted runs are not specified in the available aggregate record. This accounting limitation is made explicit in Section 3.7. The sixteen-condition grid results (Section 3.7) constitute a separate comparison; overlap with the reported total cannot be established from the aggregate summaries.

### 3.2. Angle Changes Junction Geometry and Flow Allocation Under Equal-Outlet-Pressure Conditions

**Figure 2:**
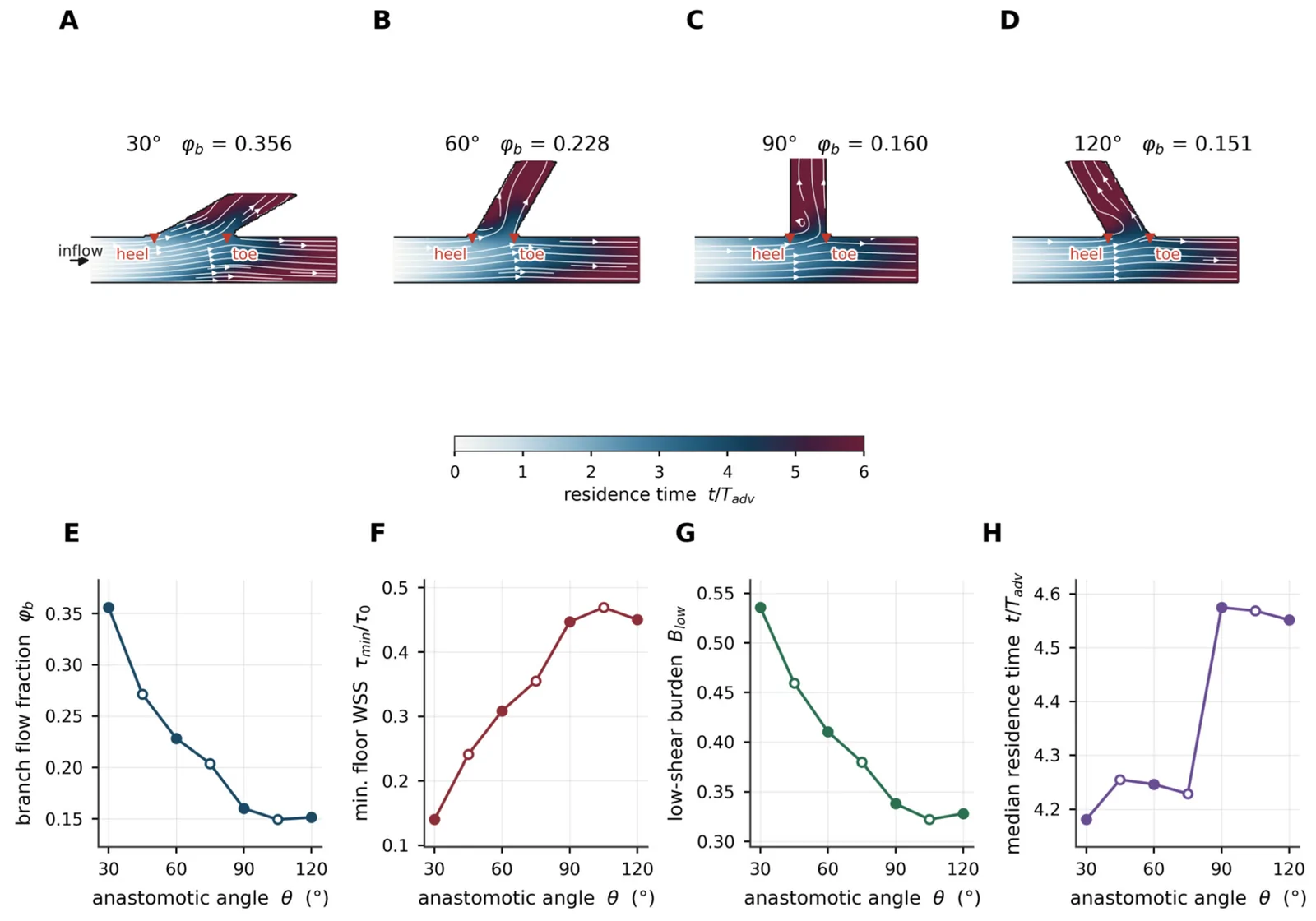
Two-dimensional angle atlas under equal outlet pressures. (**A**–**D**) Capped Eulerian age fields normalized by T = D/U_0_ at 30°, 60°, 90°, and 120°. White curves are streamlines; the wall is black, and heel and toe are marked in red. The age field includes numerical diffusion and is limited to 6T; darkest values are saturated and cannot resolve longer transport times. The field is a qualitative visualization rather than a validated particle residence-time distribution. (**E**–**G**) Branch fraction, minimum normalized signed floor shear, and signed shear-deficit burden. (**H**) The legacy display summarizes medians of the capped field; these values are excluded from the quantitative conclusions because censoring and region aggregation have not been audited. The displayed summary panels include seven angles. All displayed numerical values refer to the production grid.

Under equal static outlet pressures, the branch fraction decreased with angle from 0.356 at 30° to 0.151 at 120° (Figure 2E). Across the same range, minimum normalized signed floor shear increased from 0.140 to 0.450 (Figure 2F), while signed shear-deficit burden decreased from 0.536 to 0.328 (Figure 2G). Branch recirculation was absent at 30° and reached 7.9% of the branch region at 90°. These are production-grid observations in the planar model; the capped residence-time display in Figure 2 is qualitative.

Shallow angles therefore coincided with lower signed recipient-floor shear under equal outlet pressures, while branch recirculation increased with angle. The latter direction agrees qualitatively with Jinka et al. [5], but comparison with a published finite-volume study using a different model is contextual support, not an independent validation of this implementation.

### 3.3. Flow Matching Reduces Angle-Associated Shear Variation Under Adjusted Outlet Pressures

Matching across the angle series required the outlet bias to change from distal favoring at 30° to branch favoring at 120°. The matched conditions therefore compare different geometries under different pressure fields. The observed reduction in angular variation is conditional on this intervention.

At the approximately 0.25 target, normalized angle sensitivity of minimum signed floor shear decreased from 92.1% under equal outlet pressures to 16.1% under matching, corresponding to approximately 83% attenuation at 32 lumen nodes. Minimum normalized shear was 0.281 at 30° and 0.238 at 120° after matching. For B_low_, the corresponding sensitivities were 51.5% and 9.6%, giving approximately 81% attenuation (Table 2). These descriptive magnitudes depend on resolution; minimum-shear attenuation was approximately 74% at 64 nodes (Section 3.7).

The second target, approximately 0.21, produced the same direction of change on the production grid: attenuation was approximately 95% for minimum signed floor shear and 88% for both B_low_ and branch recirculation (Table 2). This is an additional target comparison within the same solver, not an independent numerical validation. Because its complete angle series was not repeated at 64 nodes, the larger attenuation should not be considered resolution robust.

**Figure 3:**
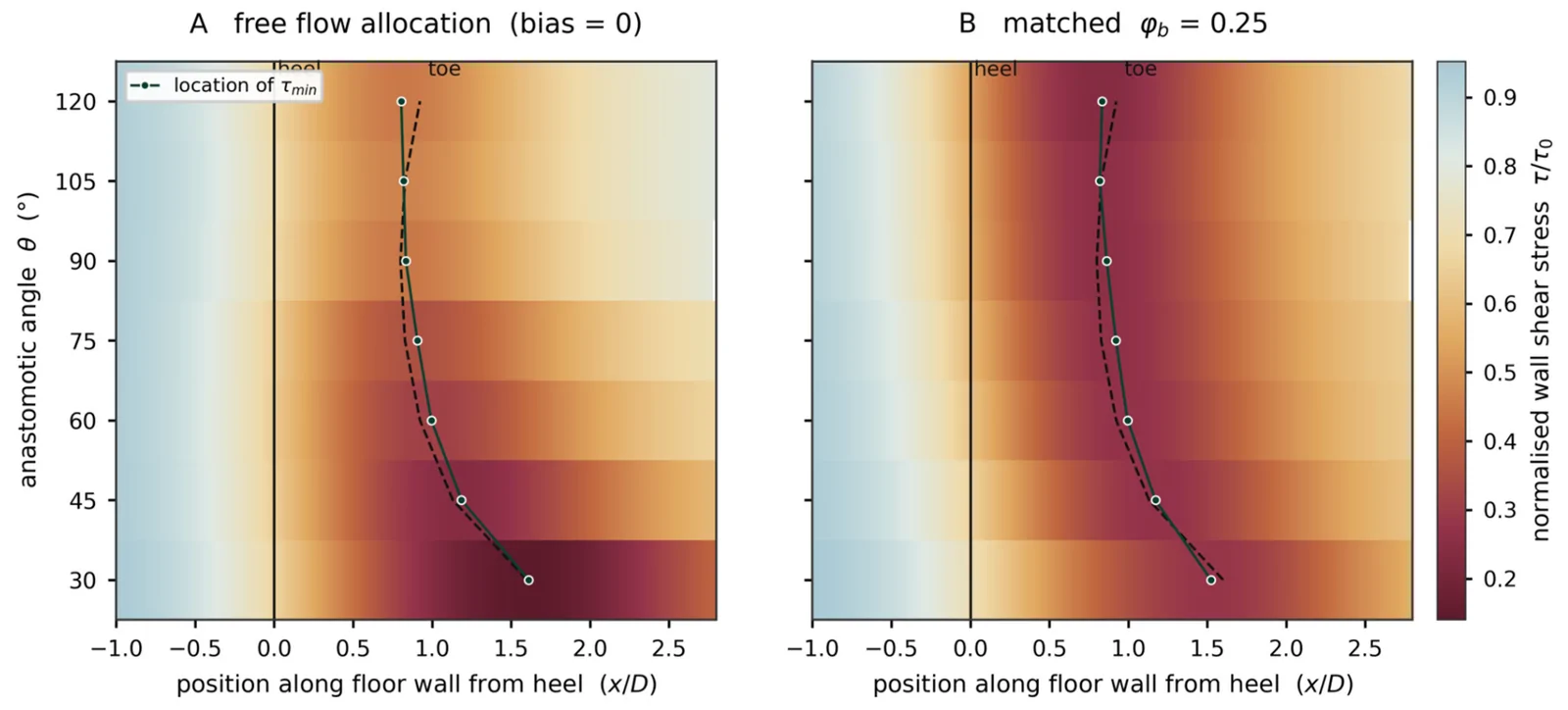
Signed recipient-floor shear under equal outlet pressures (**A**) and approximately matched branch fraction (**B**). Position is measured in recipient diameters from the heel. Each discrete band represents one simulation. The heel is marked by a solid line, the toe by a dashed curve, and minimum shear by a green track. Both panels share a color scale. The plotted quantity is signed traction, not its magnitude; matching also changes outlet pressures.

The spatial profiles provide a complementary description (Figure 3). Under equal outlet pressures, the floor-shear deficit deepened at smaller angles. At approximately the matched branch fraction, the profiles became more similar across angles, while the location of minimum shear continued to track the toe. These profiles remain conditional on the chosen outlet treatment.

### 3.4. Distal Shear Deviates from the Fully Developed Planar Reference

Under equal outlet pressures, the distal local-flow-normalized ratio D_s_ increased from 0.776 at 30° to 0.884 at 120°. These values correspond to a deficit of approximately 12–22% relative to the analytical, fully developed planar reference. They describe the sampled recovery window one to three diameters beyond the toe. Without a same-flow numerical reference or a downstream-recovery control, the deficit cannot be assigned quantitatively to junction geometry alone.

### 3.5. Regional Responses Differ Under Approximately Matched Allocation

**Figure 4:**
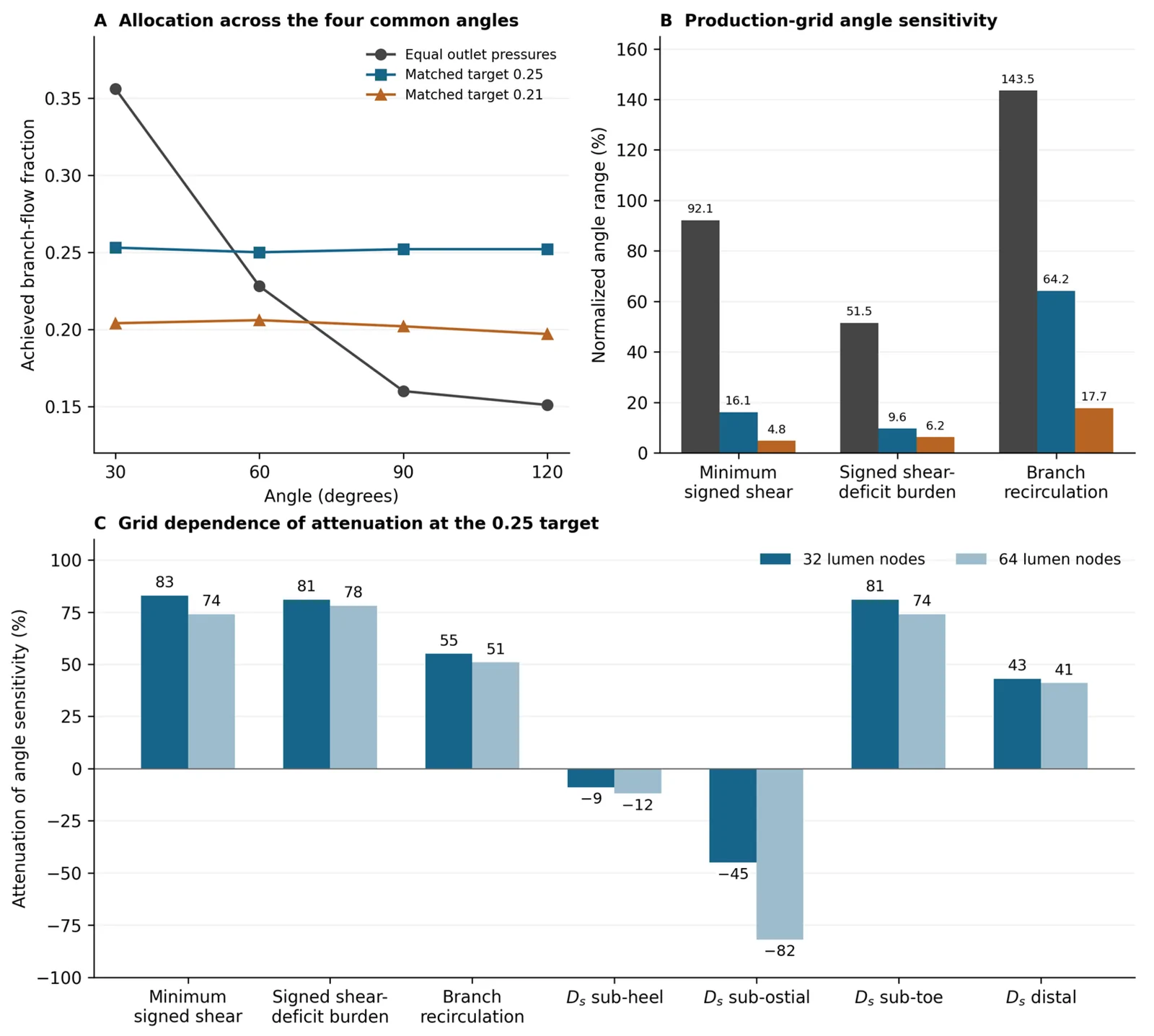
Comparisons over the four common angles, 30°, 60°, 90°, and 120°, redrawn from the reported values in Table 2 and Table 4. (**A**) Achieved branch-flow fractions. Lines connect discrete observations. (**B**) Production-grid normalized angle ranges for equal pressures and both matched targets. (**C**) Attenuation at the 0.25 target at 32 and 64 lumen nodes; negative values denote an increased angular range after matching. These descriptive percentages depend on the grid and outlet treatment and do not estimate causal mediation fractions. No new simulations are represented.

Regional analysis showed different responses to the matching intervention (Table 3, Figure 4). Sub-toe D_s_ had approximately 81% attenuation of angle sensitivity. Sub-heel and sub-ostial D_s_ instead had similar or larger normalized ranges after matching: 29.8% versus 27.3% and 27.2% versus 18.8%, respectively. Branch recirculation showed an intermediate response, with approximately 55% attenuation at the 0.25 target. These regional percentages describe the production grid.

The contrast is therefore between endpoints whose angular range decreases with the matching procedure and endpoints whose angular range persists under it. It is not a boundary-independent classification of vascular regions. The proximal reference ranged from 0.859 to 0.877 under equal outlet pressures but from 0.890 to 0.810 under matching. The latter change is 0.080 normalized units, or approximately 9% relative to 0.890, demonstrating nonlocal sensitivity to the altered downstream conditions. The available calculations do not distinguish physical upstream pressure coupling from finite-domain and boundary-treatment effects or establish that these influences are negligible.

### 3.6. Hemodynamic Structure Across the Geometry–Flow Design Space

Increasing heel and toe transition radius from 0 to 0.385D increased branch flow at every angle, by 0.055 at 30° and 0.033 at 120°. At 30°, the signed shear-deficit burden consequently rose from 0.504 to 0.583, and minimum wall shear fell from 0.166 to 0.101. At the same time, branch recirculation at 90° fell from 9.1% to 6.4%. Increasing fillet radius therefore improved branch recirculation while worsening the recipient-floor shear environment; no uniformly favorable effect was observed.

Increasing the caliber ratio from 0.60 to 1.00 also increased branch flow, from 0.143 to 0.303 at 60° and from 0.101 to 0.185 at 120°. In contrast, increasing the Reynolds number from 30 to 150 reduced branch flow. In each perturbation, changes in the recipient-floor shear environment tracked the accompanying change in flow allocation.

Across the 26 accepted production runs, branch fraction alone described 79% of the reported variation in B_low_, compared with 54% for angle alone. In the reported pooled set including matching runs (n = 40), the descriptive R^2^ values were 77% for branch fraction, 23% for angle, and 78% for both. These associations are secondary summaries of the stated design space. Exact pool membership requires the run-level inventory described in Section 3.1; the values are not used as evidence of a causal decomposition.

### 3.7. Qualitative Responses Persist with Refinement but Effect Sizes Change

Doubling lumen resolution from 32 to 64 nodes altered absolute quantities: branch fraction increased by 6–10%, minimum signed floor shear decreased by 6–23%, B_low_ changed by 3–6%, and branch recirculation changed by less than 0.01 in absolute fraction. The largest shifts occurred at shallow angles, consistent with sensitivity to oblique-wall discretization. The exercise establishes persistence of selected qualitative responses across two grids, not asymptotic convergence or grid-independent absolute values.

For the 0.25 target, minimum-shear attenuation changed from approximately 83% at 32 nodes to 74% at 64 nodes; the equal-pressure and matched sensitivities changed from 92.1% and 16.1% to 103.4% and 26.6%. B_low_ attenuation changed from 81% to 78%, and sub-toe D_s_ attenuation from 81% to 74%. Sub-ostial attenuation changed from −45% to −82%, indicating substantially increased angular range under matching at the finer grid (Table 4). Thus, the qualitative contrast between reduced distal/sub-toe variation and persisting heel/ostial variation was retained, while the magnitudes remained grid dependent. No analogous refinement conclusion is drawn for the 0.21 target. The available simulation accounting is summarized in Table 5.

### 3.8. Recipient-Lumen Flow Reversal Was Not Observed at the Simulated Reynolds Numbers

At Re ≈ 91, no node anywhere in the recipient lumen carried reversed axial velocity at any angle or transition radius; the minimum axial velocity was positive throughout. Recirculation, where present, was confined to the branch. Branch recirculation was absent at Re ≈ 30 and reached 12–13% of the branch region at Re ≈ 150.

## 4. Discussion

This study demonstrates that the apparent angle–shear relationship in a stationary two-dimensional junction changes substantially when the comparison is conditioned on approximately matched branch flow. The angular range of minimum recipient-floor shear and the sub-toe response decreased, whereas heel and ostial variation persisted under the adjusted outlet pressures. This distinction was present at both 32 and 64 lumen nodes for the 0.25 target. The result supports reporting flow allocation alongside geometry when interpreting an angle comparison within this model.

The matching intervention is central to this interpretation. It changes the relative outlet pressures, and therefore the solution throughout the domain, to obtain a similar branch fraction. The remaining angular response cannot be assigned to geometry independently of that intervention. The sensitivity of computed shear indices to boundary-condition and rheological choices has also been demonstrated in patient-specific stented coronary models [28]. Likewise, the observed 12–22% distal deficit relative to fully developed planar shear combines the response of the junction and its recovery region with numerical and boundary influences; it is not a measured geometry-only fraction. The defensible conclusion concerns how results change between two specified comparison conditions, rather than a causal partition into geometric and allocation-mediated effects.

MITOS Flow Lab contributes a transparent two-dimensional environment for posing this comparison. A planar model makes the control strategy accessible and permits systematic exploration with consistent endpoint definitions. It is nevertheless a different physical model from a cylindrical bifurcation: fully developed planar flux per unit depth scales with channel width cubed at a fixed pressure gradient, whereas cylindrical flux scales with diameter to the fourth power. Velocity profiles, junction losses, and branch partitioning therefore cannot be transferred by a simple dimensional rescaling. The methodological comparison can inform future studies without establishing that their regional response pattern will be identical.

These results provide a framework for interpreting earlier studies without requiring them to agree on a single preferred angle. Zhang et al. [1] showed in vivo that angle changes early flow allocation, with 56.5% of carotid flow entering the graft at 45° compared with 43.2% at 135°, consistent with the direction observed here. Jinka et al. [5], using pressure-based boundary conditions that allowed allocation to vary, found increasing recirculation with angle, which is reproduced by our free-allocation series. Wongchadakul et al. [6] used prescribed inlet flows, a design closer to a fixed-flow comparison, and reported smaller, non-monotonic differences between angles. Their models also differed in ostial cross-sectional area between configurations, which may itself affect allocation. Wain et al. [8] demonstrated that pulsatility changes the shear field in microanastomoses; the present results add that the interpretation of angle also depends on which hemodynamic quantity is held constant during comparison.

The wider bifurcation literature similarly relates geometry to wall-shear distributions [13,14,15]. Hydraulic-network approaches explain how distributed resistance influences allocation [17,18], while experiments and computations at moderate Reynolds numbers show that junctional inertia can make pressure–flow coupling nonlinear and angle dependent [19]. These concepts motivate controlling and reporting allocation. They do not turn the present junction into a linear circuit or establish equivalence between static numerical outlet offsets and physiological distal vascular beds.

The clinical relevance is a question for subsequent testing: does an angle comparison remain similar when flow allocation and downstream loading are measured or controlled? The present model does not establish that one angle is safer, that a shallow angle is equivalent to 90° in vivo, or that a particular regional pattern predicts intimal hyperplasia, thrombosis, or flap survival. The fillet experiment also illustrates that improving one computed endpoint can coincide with a less favorable value of another. These observations motivate an integrated assessment of geometry and flow rather than an operative angle recommendation.

Heel and ostial responses remained angle dependent under the chosen matching procedure. Although larger anastomosis studies have related local hemodynamics to intimal thickening [29], the present signed floor-shear outputs do not measure that biological process. Their spatial interpretation is limited to the planar model and to the selected regional windows.

At Re ≈ 91, no recipient-lumen flow reversal was observed in the present two-dimensional steady simulations. Recirculation, where present, was confined to the branch. This observation differs from flow patterns often reported in larger distal bypass models [29,30], but it should not be interpreted as a physiological distinction between vessel scales: the present model excludes three-dimensional secondary and helical motions that can promote separation. Experimental and three-dimensional confirmation is therefore required.

A three-dimensional extension is a future development of the program and is not part of the present implementation or evidence base. It would require its own geometry generation, numerical verification, and comparison of matched and equal-pressure conditions. Physiological pulsatility and secondary flow should then be assessed explicitly. The first validation priorities for quantitative use of the current model remain a lower-Mach comparison at a fixed physical Reynolds number, an independent two-dimensional junction benchmark, and tests of domain length and outlet implementation.

### Limitations

The model is two-dimensional, steady, rigid-walled, and Newtonian. Elastic-wall LBM formulations have been developed and compared with rigid-wall models [31]. It cannot reproduce cylindrical resistance scaling, three-dimensional secondary or helical flow, or physiological unsteady shear. Sutures and protrusions were not modeled. Numerical verification is restricted to inlet-profile checks, convergence diagnostics, conservation, and the reported grid comparisons; independent junction accuracy and finite-Mach sensitivity have not been established. The grid study did not reach a demonstrated asymptotic regime, and the 0.21 target lacks a complete refined-grid comparison. Outlet-bias and finite-domain effects are not separated from the remaining angle-associated responses. The normalized distal shear ratio lacks a same-flow numerical reference. The age field includes a cap and numerical diffusion and is used qualitatively. Finally, the aggregate simulation counts do not substitute for a reconciled run-level archive, and the computational model contains no patient data or biological validation.

These limitations constrain the numerical precision and physiological interpretation of the results. They do not change the observation that the angle comparison behaves differently under approximately matched allocation in this particular two-dimensional implementation. That observation defines the present methodological contribution; its quantitative and physiological generality remains to be tested.

## 5. Conclusions

MITOS Flow Lab provides a two-dimensional framework for comparing angle-associated hemodynamics under explicitly defined flow-allocation conditions. In the stationary model studied here, approximately matching branch flow reduced angular variation in recipient-floor and sub-toe shear, while heel and ostial variation persisted under the adjusted outlet pressures. This qualitative contrast was retained after refinement for the 0.25 target, but attenuation magnitudes and absolute shear values remained grid sensitive.

The findings support evaluating geometry together with achieved flow allocation and outlet conditions in computational angle studies. They do not quantify a boundary-independent geometric effect, establish a universal regional response in vivo, or identify an optimal surgical angle. Independent two-dimensional numerical validation is required for quantitative use; three-dimensional and physiological unsteady modeling remain future development steps.

## Figures and Tables

**Table 1:** Pre-specified recipient-floor wall regions used for regional hemodynamic feature extraction.

Region	Extent (x/D)
Proximal reference	−1.00 to −0.25
Sub-heel	−0.25 to +0.25
Sub-ostial	0 to x_toe_
Sub-toe	x_toe_ − 0.25 to x_toe_ + 0.25
Distal	x_toe_ + 1.00 to x_toe_ + 3.00

**Table 2:** Production-grid matched comparisons. Values are reported to identify the original numerical outputs, not to imply equivalent accuracy. The two matched targets are 0.25 and 0.21; tolerance is ±0.015. Angle sensitivity is calculated over 30°, 60°, 90°, and 120° only. Percentages in parentheses show the decrease in sensitivity relative to equal outlet pressures. Per-angle bias values and the complete run ledger require the original machine-readable exports; the pressure conversion is specified in Section 2.5.

Condition	θ = 30°	θ = 60°	θ = 90°	θ = 120°	τ_s,min_ Sensitivity	B_low_ Sensitivity	R_br_ Sensitivity
Free (bias 0), achieved φ_b_	0.356	0.228	0.160	0.151	92.1%	51.5%	143.5%
Matched target 0.25, achieved φ_b_	0.253	0.250	0.252	0.252	16.1% (−83%)	9.6% (−81%)	64.2% (−55%)
Matched target 0.21, achieved φ_b_	0.204	0.206	0.202	0.197	4.8% (−95%)	6.2% (−88%)	17.7% (−88%)

**Table 3:** Regional local-flow-normalized signed shear ratios and production-grid angle sensitivity. D_s_ uses the analytical planar downstream-flow reference; it does not isolate a geometry-only effect. Negative attenuation indicates increased angular range under matching.

Region	D_s_ at 30°, Free	D_s_ at 120°, Free	Angle Sensitivity, Free	Angle Sensitivity, Matched φ_b_ = 0.25	Attenuation
Sub-heel	1.032	0.796	27.3%	29.8%	−9%
Sub-ostial	0.527	0.619	18.8%	27.2%	−45%
Sub-toe	0.227	0.544	73.3%	13.9%	81%
Distal	0.776	0.884	12.8%	7.3%	43%

**Table 4:** Direct comparison of 32- and 64-node results for the four common angles under equal outlet pressures and the approximately 0.25 target. Entries are normalized angle ranges or their descriptive attenuation. The table comprises eight conditions evaluated at each of two grids; all sixteen reported condition–grid results met the stated acceptance criteria. It does not establish asymptotic convergence or test the 0.21 target.

Endpoint	Free, H = 32	Free, H = 64	Matched, H = 32	Matched, H = 64	Attenuation H = 32	Attenuation H = 64
τ_s,min_/τ_0_	92.1%	103.4%	16.1%	26.6%	83%	74%
B_low_	51.5%	52.0%	9.6%	11.6%	81%	78%
Branch recirculation	143.5%	149.8%	64.2%	73.1%	55%	51%
D_s_ sub-heel	27.3%	31.6%	29.8%	35.5%	−9%	−12%
D_s_ sub-ostial	18.8%	20.0%	27.2%	36.4%	−45%	−82%
D_s_ sub-toe	73.3%	84.4%	13.9%	21.6%	81%	74%
D_s_ distal	12.8%	14.1%	7.3%	8.3%	43%	41%

**Table 5:** Available simulation accounting. Aggregate counts are retained transparently; unassigned identities require the original run-level log.

Item	Reported Count or Status
All runs in aggregate record	62
Accepted runs in aggregate record	57
Non-accepted runs implied by totals	5
Non-accepted runs with a described failure	3; complete parameters not reported
Remaining non-accepted runs	2; identities and failure criteria unassigned
Accepted production analysis	26
Reported pooled descriptive analysis	40, including production and matching runs
62-to-analysis membership and refinement overlap	Not reconciled from the aggregate record

## Data Availability

The simulation data and source code supporting this study are available from the corresponding author upon reasonable request.
